# The true experts: codeveloping a preventive psychological intervention for postsurgical pain with patients and caregivers

**DOI:** 10.1097/PR9.0000000000001445

**Published:** 2026-04-28

**Authors:** Jana Hochreuter, Emil Kane Nissen, Cosima Locher, Jessica Lee Schleider, Shannon Bhend, Helen Koechlin

**Affiliations:** a Department of Psychosomatics and Psychiatry, University Children's Hospital, University of Zurich, Zurich, Switzerland; b Division of Child and Adolescent Health Psychology, Department of Psychology, University of Zurich, Zurich, Switzerland; c Children's Research Centre, University Children's Hospital Zurich, University of Zurich, Zurich, Switzerland; d Division of Clinical Psychology and Psychotherapy, Faculty of Psychology, University of Basel, Basel, Switzerland; e Clinical Psychology & Psychosomatics, Faculty of Psychology, University of Basel, Basel, Switzerland; f Department of Medica Social Sciences, Department of Psychology, Northwestern University, Chicago, IL, USA; g Faculty of Psychology, University of Basel, Basel, Switzerland

**Keywords:** Codevelopment, Single-session intervention, Prevention, Chronic postsurgical pain, Patients, Caregivers

## Abstract

**Introduction:**

Approximately 20% of youth undergoing major surgery develop chronic postsurgical pain (CPSP). However, preventive interventions for CPSP have yielded mixed results, partly due to the limited involvement of patients and caregivers in the intervention development process. Single-session interventions (SSI) are well-suited in this context, as they are time- and cost-efficient.

**Objectives:**

This study investigates the perioperative needs of patients and caregivers, and utilizes these insights to co-develop a preventive psychological SSI for post-surgical pain.

**Methods:**

Two rounds of focus groups were conducted with patients and caregivers to (1) gather information on the perioperative experience of patients and caregivers to develop the content of a preventive SSI, and (2) gather feedback on the draft of the intervention. Participants' experiences were analyzed applying structural content analysis. Based on these results, the content of a digital intervention was developed, which was then presented to participants for evaluation, and feedback was analyzed using participatory observation.

**Results:**

Five adolescent patients and four mothers took part in the focus groups. In total, 5 superordinate categories were identified: Consequences of context (i.e., contextual components), looking after yourself (i.e., self-care related components), limitations of healthcare system (i.e., healthcare system-based components), requests for medical staff (i.e., specific expectations directed toward providers) and child as primary focus (i.e., child-focused components). The intervention draft was well accepted by participants, and minor suggestions for improvement were implemented.

**Conclusion:**

Our study identifies core topics of importance for patients undergoing major surgery and their caregivers, and informs the development of a preventive psychological intervention.

## Abstract 2

Supplemental Digital Content is Available in the Text.

This study developed the content of a new single-session intervention targeting pain after surgery entirely based on experiences of patients and parents.

## 1. Introduction

Approximately 40% to 60% of children and adolescents who undergo surgery report moderate to severe pain during their hospital stay.^21^ If the pain persists or recurs for more than 3 months, and negatively affects quality of life, it is defined as chronic postsurgical pain (CPSP).^49^ Prevalence rates for CPSP are reported to be around 20% after major orthopedic surgery.^39,49^ It is well established that chronic pain in childhood and adolescence tends to persist into adulthood,^24,36,55^ with negative consequences on physical, psychological, social, and school domains.^17,35,54^ Although research suggests that psychosocial risk factors may contribute to the development of CPSP, previous studies have significant limitations, including small sample sizes and inconsistent definitions of CPSP.^26,40^

To date, preventive interventions to decrease the risk for CPSP have yielded mixed results.^14,29,41^ One potential reason is the limited involvement of patients and caregivers in the process of intervention content development.^2^ A growing body of evidence suggests that active engagement of patients in research processes can lead to sustainable and population-appropriate interventions, better effects, enhanced relevance to the target population, and identification of issues that researchers may not have been aware of.^10,15,16,20,22,50^ Collaboration across different stages of a project is essential: patients should not only be involved in the design of an intervention but should also be able to evaluate it.^18,23,57^

Previous qualitative research has shed light on the various challenges a surgery can create for families and has emphasized the need for perioperative psychosocial interventions.^38^ However, there are numerous barriers (eg, cost, time, access, distance to clinic) to receiving interventions,^37^ and previous research reports high drop-out rates.^7^ A low-barrier, time- and cost-effective intervention could help mitigate the impact of these issues.^7,8^ Single-session intervention (SSI) are one promising strategy to tackle these challenges. SSI are structured programs that involve a single visit or contact with a clinic, provider, or program, typically focusing on one component of empirically supported treatments.^43,44^ These programs may be digital and self-guided or delivered by trained professionals.^46^ Previous studies have shown that SSIs can be effective for various problems, such as anxiety, and increase positive health behaviors in adolescents.^28,44,46,51^ Given the high comorbidity rates of chronic pain and mental health issues,^52,53^ early interventions addressing both are essential.^40^ Single-session interventions focusing on behaviors such as relaxation or cognitive reframing skills have shown significant improvements in adults with chronic pain,^12,13^ whereas an SSI for adolescents and caregivers has reduced pain catastrophizing in both groups.^58^ For effect sizes, SSIs for psychiatric disorders including anxiety and conduct disorders in children and adolescents show only slightly smaller effects than treatments lasting an average of 16 sessions.^45,56^

This study aimed to investigate the perioperative needs of patients and their caregivers to codevelop a psychological SSI with targeted content for patients and caregivers to prevent postsurgical pain. Two rounds of focus groups were conducted with patients and their caregivers to (1) develop the content of an intervention and (2) gather feedback on the intervention draft.

## 2. Methods

### 2.1. Study design

In total, 2 rounds of semi-structured focus groups and an interview were conducted in a Swiss  university children's hospital with patients and their caregivers. The first round consisted of 2 separate focus groups for patients and caregivers, whereas the second round combined both in a single focus group and included an additional interview with a family unable to attend the focus group. Based on the experiences and needs of the participants derived in the first round, an intervention draft was developed for patients and caregivers. The drafts were tailored separately for each group and evaluated using slides summarizing the preliminary intervention content in a focus group consisting of participants from the initial focus groups. The format of the intervention—ie, SSI—was predefined; the goal of the first round was to gather patient and caregiver experiences and needs to inform its content. The study procedure was approved by the Ethics Committee of the Faculty of Philosophy, University of Zurich, Switzerland (study ID: 23.05.02).

### 2.2. Study participants

Former adolescent inpatients (aged 10–18) who recently (ie, in the 3–12 months prior) underwent orthopedic surgery and their primary caregivers were included, reflecting the fact that orthopedic procedures constitute most surgeries at the recruiting hospital and predominantly involve patients within this age group. Participants were excluded if they could not understand or speak German. Participants were recruited from the University Children's Hospital Zurich, using a search function in the medical records of patients. Patients were contacted by letter or telephone. Participation was voluntary, and patients were compensated for their time with a voucher of their choice.

Throughout the manuscript, former adolescent patients are referred to as “patients,” parents as “caregivers,” and the combined sample as “participants.”

### 2.3. Procedure

The focus groups consisted of moderated discussions centering around predefined questions^30^ and took place in the University Children's Hospital Zurich. A discussion guide was developed based on an extensive literature search, focusing on psychological pain interventions, previous qualitative studies on chronic pain, recommendations for participatory research, and guidelines for SSI development.^2–4,14,25,38,39,44^ The discussion guide contained 7 questions regarding their experiences surrounding the surgery and requests for medical staff (Table 1). The questions were discussed with patients and caregivers separately to avoid mutual influence and to ensure that the specific needs of both groups were considered. The first round of focus groups took place in August 2023. Based on the results, the intervention concept was developed in April 2024 in collaboration with a team of SSI experts from Northwestern University Chicago (under the supervision of J.L.S.).

**Table 1:** Questions for focus groups.

Patients
1. Would a program that prepares children and their caregivers to deal with pain and anxiety after surgery be helpful? Why/why not?
2. What were setbacks or stressful moments before surgery?
3. What was the most difficult part surrounding the surgery?
4. What were you not prepared for or what surprised you?
5. What advice would you give patients of the same age in this situation to help them better deal with difficult moments or feelings surrounding the surgery?
6. Are there situations in which you have successfully dealt with difficult feelings? How did you do that?
7. What do you need from the medical staff? What should they pay particular attention to and what do they absolutely need to know?
Caregivers
1. Would a program that prepares children and their caregivers to deal with pain and anxiety after surgery be helpful? Why/why not?
2. What were setbacks or stressful moments before surgery?
3. What was the most difficult part surrounding the surgery?
4. What were you not prepared for or what surprised you?
5. What advice would you give caregivers in this situation to help them deal better with difficult moments or feelings surrounding the surgery?
6. Are there situations in which you have successfully dealt with difficult feelings? How did you do that?
7. What do you want from the medical staff? What should they pay particular attention to and what do they absolutely need to know?

Translated from Swiss German to English.

The topics derived from the first round of focus groups were grouped into the following intervention components: psychoeducation, peer testimonial, activity, and action plan, which are typically included in SSIs.^44^ The specific content was adapted to the respective age (ie, children and adolescents), and recipient group, so that patient- and caregiver-specific needs could be covered. In June 2024, the proposal was presented to and evaluated by participants from the first round of focus groups, this time bringing both groups together to encourage interaction.

The discussions were audio-recorded and transcribed verbatim for the first round. In the second round, information was audio-recorded and documented with the help of participatory observation of 2 independent researchers. Data transcription was anonymized. Quotations from the discussions have been translated from Swiss German to English. We also collected socio-demographic information from participants.

### 2.4. Qualitative analysis

Two distinct approaches were used to analyze the first and second rounds of focus groups. The goal of the first round was to identify the needs of participants to inform the content of the intervention. The purpose of the second round was to collect feedback on the intervention draft created based on the first round's findings.

**Figure 1.**
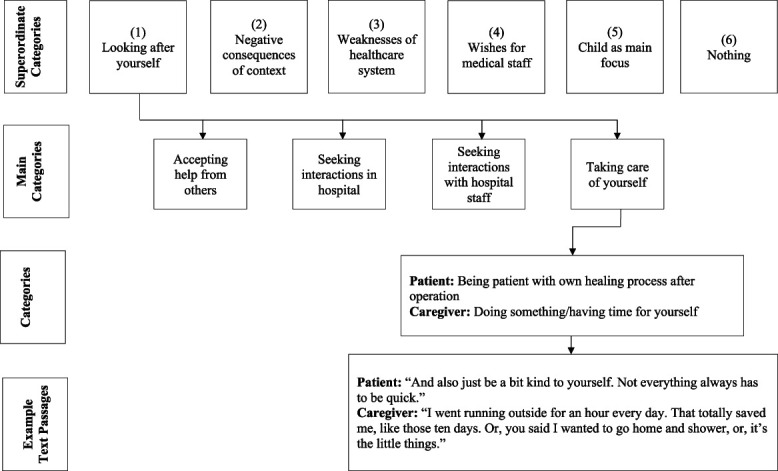
Coding procedure.

Applying structural content analysis based on Mayring,^31^ the first round of focus groups (questions provided in Table 1) was coded and analyzed using the online software QCAmap (www.qcamap.org),^19^ with 2 independent raters (J.H. and S.B). An inductive data-driven approach was applied, which enables the exploration of core themes in a phenomenon with limited existing theory or research literature.^27^ A multistage analytic process was conducted^31^ (Fig. 1): first, the transcript was worked through line by line. Text passages that aligned with the corresponding research question were coded with appropriately defined categories. After 20% of the material had been analyzed, the category system was revised in a consensus meeting including the project leader (H.K.), which resulted in minor adjustments. Subsequently, the entire dataset was analyzed applying identical criteria (ie, category definition and level of abstraction). The final list of categories was organized into main categories. Intercoder agreement was assessed by 2 authors (H.K. and C.L.), who reviewed the text material applying all content-analytical rules. Disagreements were solved in further consensus meetings including the project leader. Finally, frequency analyses of the category occurrences in the text material were conducted.

For the second focus group and the additional interview, an adapted form of participatory observation^6,32,42,48^ was chosen. The observers' documentations were later analyzed to extrapolate core themes. These were then assessed by the first author (J.H.) and adjusted until a consensus was reached among the first author and the observers. These insights were used to revise the intervention draft. This approach was chosen because the second round of focus groups solely aimed to gather of feedback on the intervention draft. To ensure an unbiased review of the feedback provided by the focus group, we included independent researchers to act as participatory observers.

## 3. Results

### 3.1. Sample characteristics

Five adolescent patients (aged 14–16 years) took part in the focus group, 20% identified as female and 80% as male. Two of the patients are twins. All patients underwent major surgeries that required an inpatient stay. The participating caregivers were 4 mothers. See Table 2 for more information on sample characteristics.

**Table 2:** Descriptive statistics of samples.

Patients	N = 5
Age, M (SD)	15 (1)
Gender, n (%)	
Male	4 (80%)
Female	1 (20%)
Current education level, n (%)	
Secondary school	3 (60%)
10th year of school	1 (20%)
Special needs school	1 (20%)

Caregivers	N = 4
Age, M (SD)	52.5 (3.79)
Gender, n (%)	
Male	0 (0%)
Female	4 (100%)
Highest completed education level, n (%)	
Apprenticeship	2 (50%)
High school	1 (25%)
PhD or higher	1 (25%)

### 3.2. Identification of perioperative needs and requests of patients and caregivers

In total, 472 text passages were identified and condensed into 106 categories. These categories were further organized into 17 main categories and grouped into 5 superordinate categories (Table 3). Because of the large number of overlapping themes, identical categories of the 2 groups were combined. An overview of all superordinate categories, main categories, and categories is provided in the supplement, alongside the origin of each category from the subsample and the number of quotations per category (eTable 1, http://links.lww.com/PR9/A404). In the following section, the 5 superordinate categories are delineated (see also Table 3). The order of presentation reflects the number of quotations within each category, aligning with Mayring's approach to complement the qualitative insights with quantitative support.^31^ However, as frequency does not necessarily equate to salience, all categories were given equal consideration in developing the intervention.

(1) Negative consequences of context (contextual components): physical, mental, and environmental challenges in the hospital

**Table 3:** Superordinate categories with corresponding main categories.

Superordinate categories	Main categories
1. Looking after yourself	Accepting help from others (C)
	Seeking interactions in hospital (PC)
	Seeking interactions with hospital staff (PC)
	Taking care of yourself (PC)
2. Consequences of context	Lack of control over context (PC)
	Negative impact of hospital context on physical and mental well-being (PC)
	Physical consequences of surgery/context (P)
	Worries and fears around surgery and pain (PC)
3. Weaknesses of health care system	Gathering information yourself (C)
	Lack of information (C)
	Lack of support (C)
	Not feeling heard and seen by hospital staff or parents (P)
4. Wishes for medical staff	Making one feel heard and seen (PC)
	Providing information (PC)
5. Child as main focus	Extent of child's needs (C)
	Taking care of child's needs (PC)
	Taking child's needs seriously (PC)

C, caregivers; P, patients.

Patients emphasized the negative physical and mental consequences resulting from their hospital stay. In addition to the pain caused by the surgery, stressful situations and sleep issues were common. For both patients and caregivers, noise was an important issue that had caused them problems while in the hospital, leading to stress and negatively affecting their sleep.

Patient: After I was hospitalized, I was in a really loud room for quite a long time. And that was just extremely stressful for me […].Because when it's so loud that you can't distract yourself, it's quite difficult.

Circumstances over which the participants had no control were also described as stressful. These included COVID-19 restrictions and long waiting periods. The surgery also triggered worries and fears: although the patients were more afraid of the surgery itself, the caregivers were most worried when they had to watch their child experiencing pain.

Mother: You know how in movies people use a voodoo doll, to torture someone or, like maltreat them over distance somehow, and children are like such voodoo dolls I think for the caregivers. And the smaller they are, the worse, I think.

Caregivers felt cramped in the hospital room and felt they had no space for themselves. They had to put away their folding beds immediately after getting up in the morning, and despite their important role, they felt unwelcome in the hospital due to the lack of space.

Mother: But for me, it's also about giving space. For the caregivers. […] The space you have is just a chair! And for such a long time. And you don't even have a hook, let alone a closet.

(2) Looking after yourself (self-care related components): importance of self-care, patience, and social interactions

The participants considered self-care to be crucial. For the caregivers, this meant accepting help, both hands-on and emotional, from their social network. They highlighted the importance of taking care of themselves to fulfil their crucial caregiving role.

Mother: I went running outside for an hour every day. That totally saved me, like those ten days. Or, I wanted to go home and shower, or, it's the little things.

Patients emphasized distraction, and podcasts, books, games, or taking a nap helped them. However, one patient also explained how they had to hold back from social media to avoid constantly seeing what they were missing out on. In addition to distraction, the patients specified that acceptance and patience are essential.

Patient: And also just be a bit kind to yourself. Not everything always has to be quick.

Both groups appreciated interactions in the hospital, both with other patients and other caregivers, not going through this time alone was considered valuable. Patients also added the positive effect of humor, as it made everyday life easier.

(3) Limitations of health care system (health care system–based components): lack of information, support, and validation in the hospital

Lack of information was a major difficulty mentioned by all participants. There was a lack of information about what to expect during an in-patient stay, but also a lack of realistic preparation for patients regarding the surgery and healing process, and for caregivers regarding their important role even while their child was in the hospital. Caregivers stated that they had to organize everything themselves from the outset, such as finding out what aids were available in the hospital. They also emphasized that they had to come up with solutions for their child's problems, and that they felt helpless and alone. Further, they felt like they did not receive enough support from the hospital staff.

Mother: And you're actually like non-existent, but then you still have a leading role. It's just a strange contradiction.

Meanwhile, patients claimed that they often did not feel like the primary focus of caregivers and medical staff. Formally addressing the medical staff created too much distance, and patients also felt left out of conversations between their caregivers and physicians, and unable to speak for themselves.

Patient: I don't know, [I wish] that the doctors don't just talk to the caregivers, but also to the child. So, yes: “Has [name] eaten a lot today?” Then you think to yourself: “Hello, [name] to doctor. [Name] here. You can ask [name] yourself.” And doctors, especially […] before surgery, you always have a consultation. And then caregivers are often given a lot of space, but the child can't ask questions. Because I think the child has to approve of the surgery to a certain extent and the child has to have all their questions answered, because the child is the one who has to deal with the consequences.

(4) Requests for medical staff (specific expectations directed toward medical staff): desire for proactive support and validation

Participants suggested that medical staff should behave in ways that make them feel heard and seen. To achieve this goal, patients emphasized that staff should show interest in them as individuals rather than solely as patients, including engaging in topics beyond the hospital context (eg, hobbies), which was perceived as fostering closeness. According to both groups, patients' needs should be taken seriously, including the timely management of any level of pain reported. Caregivers emphasized the importance of being proactively informed, for instance about unexpected waiting times, including clear explanations and anticipated time frames.

Mother: […] what I would have appreciated is … the surgery ended up taking 2.5 hours longer than it should have. If during that time someone had informed me: Is it taking longer because they started later? Is it taking longer because she’s now paralyzed? Is it taking longer because something else happened—just why is it taking longer, and what is the approximate timeframe?

Caregivers reported that they should feel valued, the medical staff should show more understanding for their situation, and offer them access to counselling and psychological support.

Mother: Our daughter was offered psychological help, which she didn't want to take up at that moment. It occurred to me straight away that it might have been more effective for the situation if I had been able to talk for half an hour and […] be more confident or trusting or whatever.

(5) Child as primary focus (child-focused components): need for validation and recognition as the main individual receiving treatment

Caregivers stressed the surprisingly large extent of the child's needs, ranging from sleep to digestive problems. They were surprised at the amount of things they themselves had to take care of, and how essential their support was for their child. Both groups agreed that hospital clown visits, if desired by the patient, can be helpful, and that familiar objects from home can make everyday hospital life easier.

Patient: I took a blanket from home on day three, for example, which-, so-, just things that you know from home or from everyday life that could help you. Somehow your own clothes are comfortable or yes, just things that you know or somehow music that you like to listen to or things like that, that always helps.

Both patients and caregivers underlined the importance of taking patients' needs seriously. Medical staff should proactively ask about their needs and try to fulfil them. According to the patients, the division of rooms according to age groups should also be respected, and the privacy of individual patients needs to be protected within the room.

Patient: And also, that […] the nursing staff is more responsive to the individual needs of the patient. And you also have to do this more with children than with adults. You have to do it more with everyone, but especially with children.

### 3.3. Development of intervention concept

Based on the content of the qualitative analysis, an intervention concept for each group (patients and caregivers) was developed. The intervention will be digital, self-administered, and consist of 4 elements, based on SSI best practices: (1) a psychoeducation component covering details about the hospital stay, basic pain models, and how pain can be influenced; (2) peer testimonials, including those of patients and caregivers in similar situations, as well as advice from the participants of the present study; (3) coping strategies and potentially helpful resources rated by users; and (4) an individualized action plan based on these ratings. All components are intended to be integrated into a single website, ensuring low-barrier access. More details will be published in a separate manuscript upon completion of intervention development.

The intervention concept was well received by all participants. Accessibility was emphasized, ensuring that patients and caregivers, regardless of their condition, can profit from it. The participants also agreed that it would be beneficial to have different versions, depending on the age of the patient. In addition, the desire for both a text and a video format was expressed, further suggesting the desire for variety within the intervention. Several aspects regarding peer testimonials were mentioned: although they should not induce anxiety, they should also not lead to an underestimation of the significance of the situation ahead. Similarly, it was mentioned that the peer testimonials should be heterogeneous, preparing patients and caregivers for the fact that it is not possible to accurately predict the course of the individual recovery process. Finally, it was noted that it is important to not only think of the physical recovery process but to take the psychological aspects seriously, both for patients and for caregivers.

Mother: Maybe add a check-up [on] what it has done to the soul, it takes years to process that, even as caregivers. […] It is not only a piece of meat; it is a human being.

Finally, participants mentioned that it would be important to receive the intervention before the surgery, together with a reminder after surgery.

## 4. Discussion

The current study aimed to investigate the perioperative needs of former patients and their caregivers in the hospital setting and beyond, and, based on those outcomes, develop and evaluate the content of a psychological preventive intervention. Our findings indicate that families encountered physical, mental, and organizational challenges, as well as a feeling of lack of support and information. Patients and caregivers were open to a psychological intervention to help them deal with challenges during the perioperative time. The intervention draft based on the results of the first round of focus groups is digital, self-guided, includes components based on best practices in SSI design,^43^ and will be reported elsewhere in detail upon completion.

Families face a multitude of challenge in the hospital including emotional challenges, such as anxiety related to the surgery itself, and beliefs of caregivers that they were unable to provide adequate psychosocial support to their children.^38^ In addition, the fear of painful procedures and the burden of the hospital context in general are often reported,^1^ as is the lack of information and involvement of patients.^9^

Participants in our study emphasized self-care in the form of being patient with their own healing process, accepting help from others, and taking some time out. Further, it is crucial to prioritize patients, and giving them the space to express their needs and opinions about the care they receive.^5^ After all, youth have a right to participate in research and decisions that inform their medical care.^59^ Although some countries have advanced procedures to include patients and caregivers in research (such as Canada),^2^ this is currently not the case in Switzerland.

Although some topics identified in our study are contextual and difficult to change, others present as modifiable targets for psychosocial interventions. The SSI developed based on these results aims to be time- and cost-efficient. Based on the 4 common elements of SSI,^44^ it aims to offer participants realistic preparation for the perioperative period, and equipping them with coping strategies for challenging situations. The intervention will be adapted based on the specific feedback gathered and then tested in a randomized controlled trial. Future studies could investigate whether interventions focusing on medical staff and informed by patient and caregiver report may be beneficial.

### 4.1. Strengths and limitations

To the best of our knowledge, this is the first study to codevelop a preventive SSI in the pediatric perioperative setting with the intervention content relying on patients' and caregivers' experiences and needs. Second, the qualitative approach enhanced an in-depth exchange and understanding of families' experiences and needs. Third, the fact that the feedback on the intervention gathered was analyzed by the independent observers minimized the likelihood of bias. Finally, the patients included in this study experienced a range of orthopedic surgeries as well as variable pain levels and postoperative recovery courses; notably, no surgery-specific needs emerged.

Despite these strengths, our study also comes with several limitations: first, the study was conducted at one single children's hospital in Switzerland, which limits external validity. Second, the included sample lacked sufficient diversity, especially in age and race, which might affect the generalizability of the findings across different demographic groups. Third, although we planned to include patients between the ages of 10 and 18, the small age range of our actual sample indicates a possible bias in recruitment. Fourth, the participating caregivers were exclusively mothers. Fifth, although the selection of questions is grounded in extensive literature research, the effects of researcher allegiance cannot be completely ruled out.^11,33^ Sixth, the interval of up to 12 months since surgery could have contributed to recall bias among the participants.^34,47^ Finally, although we included orthopedic surgeries only, patients' needs may vary across different procedures. However, the intervention content is kept broad and focuses on the general hospital experience rather than being surgery specific.

## 5. Conclusion

This study examined the needs and requests of adolescent patients and their caregivers in the perioperative setting. Topics ranged from self-care to dealing with external and unchangeable factors and were grouped into a psychological SSI format. The low-threshold, time- and cost-effective SSI draft was then evaluated by the participants, and their feedback was used to refine the intervention draft. Finally, and importantly, this study demonstrates how collaborating with patients and caregivers can lead to the development of efficient, relevant, credible, and accessible intervention content. It is our hope that this study will raise awareness about the importance of patient and caregiver's collaboration and active participation in research, even in settings where this involvement is currently lacking.

## Disclosures

The authors have no conflict of interest to report.

## Supplemental digital content

Supplemental digital content associated with this article can be found online at http://links.lww.com/PR9/A404.
